# Supplementary material for: Resolving the full spectrum of human genome variation using Linked-Reads
Source: Genome Res. 2019 Apr;29(4):635–45. doi: 10.1101/gr.234443.118 (PMC6442396; doi:10.1101/gr.234443.118)
Supplement: Supplemental Material [file supp_gr.234443.118_Supplemental_longranger-2.2.zip › longranger-2.2.tar.bz2!/lr2.2-public/lib/stan/cmdstan/stan/doc/api/html/do__bfgs__optimize_8hpp_source.html]

Stan: src/stan/common/do\_bfgs\_optimize.hpp Source File


|  |  |
| --- | --- |
|  | Stan  2.5.0  probability, sampling & optimization |


- Main Page
- Related Pages
- Namespaces
- Classes
- Files

- File List
- File Members

All Classes Namespaces Files Functions Variables Typedefs Enumerations Enumerator Friends Macros Pages

- src
- stan
- common

do\_bfgs\_optimize.hpp

Go to the documentation of this file.

1 #ifndef STAN\_\_COMMON\_\_DO\_BFGS\_OPTIMIZE\_HPP

2 #define STAN\_\_COMMON\_\_DO\_BFGS\_OPTIMIZE\_HPP

3

4 #include <fstream>

5 #include <iostream>

6 #include <iomanip>

7 #include <vector>

8

9 #include <stan/gm/error\_codes.hpp>

10

11 #include <stan/common/do\_print.hpp>

12 #include <stan/common/write\_iteration\_csv.hpp>

13 #include <stan/common/write\_iteration.hpp>

14

15 namespace stan {

16

17  namespace common {

18

19  template<typename ModelT,typename BFGSOptimizerT,typename RNGT,

20  typename StartIterationCallback>

21  int do\_bfgs\_optimize(ModelT &model, BFGSOptimizerT &bfgs,

22  RNGT &base\_rng,

23  double &lp,

24  std::vector<double> &cont\_vector,

25  std::vector<int> &disc\_vector,

26  std::fstream\* output\_stream,

27  std::ostream\* notice\_stream,

28  bool save\_iterations,

29  int refresh,

30  StartIterationCallback& callback) {

31  lp = bfgs.logp();

32

33  if (notice\_stream)

34  (\*notice\_stream) << "initial log joint probability = " << lp << std::endl;

35  if (output\_stream && save\_iterations) {

36  write\_iteration(\*output\_stream, model, base\_rng,

37  lp, cont\_vector, disc\_vector);

38  }

39

40  int ret = 0;

41

42  while (ret == 0) {

43  callback();

44  if (notice\_stream && do\_print(bfgs.iter\_num(), 50\*refresh)) {

45  (\*notice\_stream) << " Iter ";

46  (\*notice\_stream) << " log prob ";

47  (\*notice\_stream) << " ||dx|| ";

48  (\*notice\_stream) << " ||grad|| ";

49  (\*notice\_stream) << " alpha ";

50  (\*notice\_stream) << " alpha0 ";

51  (\*notice\_stream) << " # evals ";

52  (\*notice\_stream) << " Notes " << std::endl;

53  }

54

55  ret = bfgs.step();

56  lp = bfgs.logp();

57  bfgs.params\_r(cont\_vector);

58

59  if (notice\_stream && (do\_print(bfgs.iter\_num(), ret != 0 || !bfgs.note().empty(),refresh))) {

60  (\*notice\_stream) << " " << std::setw(7) << bfgs.iter\_num() << " ";

61  (\*notice\_stream) << " " << std::setw(12) << std::setprecision(6)

62  << lp << " ";

63  (\*notice\_stream) << " " << std::setw(12) << std::setprecision(6)

64  << bfgs.prev\_step\_size() << " ";

65  (\*notice\_stream) << " " << std::setw(12) << std::setprecision(6)

66  << bfgs.curr\_g().norm() << " ";

67  (\*notice\_stream) << " " << std::setw(10) << std::setprecision(4)

68  << bfgs.alpha() << " ";

69  (\*notice\_stream) << " " << std::setw(10) << std::setprecision(4)

70  << bfgs.alpha0() << " ";

71  (\*notice\_stream) << " " << std::setw(7)

72  << bfgs.grad\_evals() << " ";

73  (\*notice\_stream) << " " << bfgs.note() << " ";

74  (\*notice\_stream) << std::endl;

75  }

76

77  if (output\_stream && save\_iterations) {

78  write\_iteration(\*output\_stream, model, base\_rng,

79  lp, cont\_vector, disc\_vector);

80  }

81  }

82

83  int return\_code;

84  if (ret >= 0) {

85  if (notice\_stream)

86  (\*notice\_stream) << "Optimization terminated normally: " << std::endl;

87  return\_code = stan::gm::error\_codes::OK;

88  } else {

89  if (notice\_stream)

90  (\*notice\_stream) << "Optimization terminated with error: " << std::endl;

91  return\_code = stan::gm::error\_codes::SOFTWARE;

92  }

93  if (notice\_stream)

94  (\*notice\_stream) << " " << bfgs.get\_code\_string(ret) << std::endl;

95

96  return return\_code;

97  }

98

99  }

100

101 }

102 #endif

103

write\_iteration\_csv.hpp

stan::gm::error\_codes::SOFTWARE

**Definition:** error\_codes.hpp:15

stan::gm::error\_codes::OK

**Definition:** error\_codes.hpp:11

stan::common::do\_print

bool do\_print(const int n, const bool special, const int refresh)

Indicates whether it should print on the current iteration.

**Definition:** do\_print.hpp:25

error\_codes.hpp

stan::common::write\_iteration

void write\_iteration(std::ostream &output\_stream, Model &model, RNG &base\_rng, double lp, std::vector< double > &cont\_vector, std::vector< int > &disc\_vector)

**Definition:** write\_iteration.hpp:19

write\_iteration.hpp

stan::common::do\_bfgs\_optimize

int do\_bfgs\_optimize(ModelT &model, BFGSOptimizerT &bfgs, RNGT &base\_rng, double &lp, std::vector< double > &cont\_vector, std::vector< int > &disc\_vector, std::fstream \*output\_stream, std::ostream \*notice\_stream, bool save\_iterations, int refresh, StartIterationCallback &callback)

**Definition:** do\_bfgs\_optimize.hpp:21

do\_print.hpp


---

[ Stan Home Page ]

*© 2011–2014,
Stan Development Team.*
